# Wheatgrass (*Triticum aestivum*) growth and nutrient composition in Aquaponics with African catfish (*Clarias gariepinus*) using Einheitserde and coconut: vermiculite substrates

**DOI:** 10.1007/s11356-025-36666-z

**Published:** 2025-06-27

**Authors:** Ulrich Knaus, Christoph Hiller, Lu Xu

**Affiliations:** Faculty of Agricultural and Environmental Sciences, Department of Aquaculture and Sea-Ranching, University of Rostock, Justus-von-Liebig-Weg 6, D-18059 Rostock, Germany

**Keywords:** Circular economy, Waste valorisation, Plant secondary metabolites, Dietary supplements, Integrated systems, Aquaponics, Peat alternatives

## Abstract

Wheatgrass (*Triticum aestivum*)  was cultivated to identify biochemical compounds and nutrients for use as dietary supplements as a new method of aquaponic plant utilisation. *T. aestivum* was irrigated with aquaculture process water from an extensive aquaculture unit (EAU) and an intensive aquaculture unit (IAU) of African catfish production (*Clarias gariepinus*) for nine days after transplanting (DAT), and plant growth was compared to a control using commercial fertiliser. Plants were cultivated in pots filled with the standard media “Einheitserde” (80% white peat, 20% clay) and a coconut and vermiculite substrate (50:50, C/V). The best plant growth was observed in *T. aestivum* irrigated with IAU and cultured in the C/V substrate, while no influence of the different irrigation solutions was found in moisture content, crude protein, crude lipid, ash, or crude fibre. The C/V substrate functioned as a nutrient reservoir (e.g. P, K, Mg, B, Fe, Mn), except for nitrogen, which originated in substantial proportions from the IAU process water. *T. aestivum* grown in the C/V substrate showed no difference in mineral content between EAU and IAU, while process water from the IAU only improved growth parameters. Vitamin B_6_ was the highest in *T. aestivum* when cultured in IAU and C/V substrate. Folic acid (B_9_) reached acceptable levels for human consumption, and vitamin B_12_ achieved the amount of 96.5% of the recommended concentration. Aquaponics cultivation of *T. aestivum* is possible with *C. gariepinus* production and under substitution of the standard media “Einheitserde” with a coconut and vermiculite substrate (50:50), to reduce the amount of peat in aquaponics farming.

## Introduction

Aquaponics aims to achieve sustainable plant production by recycling nutrient-rich aquaculture wastewater, thereby decreasing the reliance on synthetic fertilisers (Yep and Zheng 2019). Therefore, plants with low nutrient requirements have been tested such as fast growing culinary or medicinal herbs with high economic value, including mint (*Mentha* spp.; Roosta and Sajjadinia 2010; Rakocy 2012) or basil (*Ocimum basilicum*; Ferrarezi and Bailey 2019; Harika et al. 2024). As a new approach, secondary plant metabolites could also be used as extracts for the production of pharmaceuticals, cosmetics or food supplements and thus complete the aquaponic value chain depending on the quantity of product (Yahya et al. 2018; Faccio 2020; Chiocchio et al. 2021). The extraction of plant ingredients offers an alternative to the highly regulated EU organic certification, which prohibits the sale of live plants that have come into contact with aquaculture effluents (Fruscella et al. 2021). A plant that grows quickly in aquaponics pot culture and from which extracts can be used for food is wheat (*Triticum* spp.) which has been tested only to a limited extent before.

Young wheat plants (*Triticum aestivum*, family: Poaceae, sweet grasses), which require 6–10 days to germinate, are referred as “wheatgrass” (Benincasa et al. 2019). Wheat is considered the most important dietary “bread” crop in temperate countries and as the main carbohydrate source for about 40% of humans (Heyne 1987; Kashyap et al. 2022). For 2021/22, the global wheat production was projected to be ≈ 770 million tons, and demand is expected to increase by 60% in 2050 (FAO 2022; Mittal 2022). In the USA, wheatgrass (*T. aestivum*) and barley (*Hordeum vulgare*, Poaceae) showed sales increase of 131.9% from 2015 to 2016 (total sales $5,770,618 of both crops), as it used as dietary supplements for detoxification, digestive support, and immune system benefits as “superfoods” or “supergreens” (Smith et al. 2017; van den Driessche et al. 2018). The global wheatgrass market, valued at approx. 62.4 million US$ in 2023, is projected to grow by ≈ 6% (CAGR: Compound Annual Growth Rate) to reach 114.4 million US$ by 2033 (FMI 2024), driven by products such as smoothies, protein or fruit shakes, and dietary supplements (Adrianos et al. 2017). In this process, the young shoots (8–10 days old) are juiced and either consumed directly, added to smoothies, or taken as capsules or in tablet form (Adrianos et al. 2017). Wheatgrass is high in minerals, amino acids, vital enzymes, and vitamins, it acts as an anti-allergic compound and appears to have an anti-cancer effect (Boukid 2021). Wheatgrass juice may have high levels of B vitamins, which are particularly important in vegetarian and vegan diets e.g. with vitamin B_12_ as an anti-anaemic factor (Lae and Oo 2014). *T. aestivum* has been successfully tested in nutrient-film-technique (NFT) systems using artificial nutrient solutions (Mackowiak et al. 1989; Page and Feller 2013) and in aquaponics combined with African catfish (*C. gariepinus*; Xu et al. 2022). Thus, cultivation under controlled conditions is feasible for human consumption and shows great promise for use as a dietary supplement.

Standard media or “Einheitserde” (“Fruhstorf soil” or “Nullerde”) originally consisted mainly of decomposed raised bog peat (94%) and clay (Günther 1999) with very good plant cultivation properties such as low pH value, low nutrient content, low microbial activity and good water and air supply (Schmilewski 2015). In the EU, peat (mainly *Sphagnum* spp.) is the most important organic substrate with about 75% utilisation (2013; Schmilewski 2017) and has been used for greenhouse cultivation and as a soil conditioner (Joosten and Clarke 2002; Savvas and Gruda 2018). However, peat extraction releases carbon dioxide (CO_2_), methane (CH_4_), and smaller amounts of nitrous oxide (N_2_O), which increases global warming (Gorham 1991; Nykänen et al. 1996; Reumer et al. 2018). Therefore, peat-based growth media should be replaced by wood fibres, composted bark, biogenic waste, maize and coconut fibres, or humus-mineral-complexes (Eymann et al. 2015; Barrett et al. 2016; Knaus et al. 2024).

Coconut fibre (*Cocos nucifera*) has peat-like properties such as good water and air retention (Barrett et al. 2016; Savvas and Gruda 2018) and has been recommended as an alternative substrate for tomato production (Osvalde et al. 2021). In aquaponics, coconut fibre has already been successfully used for lettuce (*Lactuca sativa*) in combination with the Nile tilapia (*Oreochromis niloticus*; Jordan et al. 2018). Cocopeat (coir fibre pith) was evaluated as particularly suitable for the hydroponics of cucumbers (*Cucumis sativus*; Singh et al. 2018). However, coconut fibre also has negative properties in the tropical environment due to excessive planting of palm tree monocultures as well as high water requirements, processing and transport and should itself be proportionally reduced in the substrate (Eymann et al. 2015).

Vermiculite, a layered silicate (Mg, Al, and Fe), has good buffering, aeration and water retention properties and can store and release nutrients such as magnesium and potassium due to its high cation exchange capacity (Resh 2012; Savvas and Gruda 2018). Vermiculite showed a very high germination success of 98.6% in tomato seeds (Hota and Arulmozhiselvan 2017). In aquaponics with an attached living wall (Terapia Urbana), basil (*Ocimum basilicum*), and mint (*Mentha viridis*) grew better in vermiculite compared to that in coconut fibre (Khandaker and Kotzen 2018). Vermiculite alone does not provide an organic source of nutrients and should therefore be used in combination with other substrates, such as peat, or with perlite for improved aeration (Maucieri et al. 2019).

African catfish (*Clarias gariepinus*) was introduced as a new species in aquaponics about 10 years ago and was successfully tested in co-cultivation with water spinach (*Ipomoea aquatica*; Endut et al. 2010), amaranth (*Amaranthus* spp.; Mamat et al. 2016), and basil (*Ocimum basilicum*; Knaus and Palm 2017). African catfish production has increased in the past few years to about 918 t in Mecklenburg-Western Pomerania Northern Germany due to very good growth characteristics (Brämick 2019) and is enjoying increasing demand from customers.

The present study tested the growth of winter wheat *T. aestivum* (Poaceae) as a crop with low nutrient requirements (Somerville et al. 2014) under semi-continuous aquaponics [*s.l.*] farming conditions after Palm et al. (2024) in horticulture. The aim of the study was to identify an alternative substrate mixture to replace the standard media “Einheitserde” with a high peat content. The hypothesis to be tested was that a mixture of 50% coconut substrate and 50% vermiculite, supplemented with liquid waste of varying nutrient composition from African catfish aquaculture, would support the same growth and nutrient content in *T. aestivum* as when cultivated in 100% “Einheitserde” substrate. Wheat plants were cultured in garden pots in a standard media “Einheitserde” after Fruhstorfer (80% white peat, 20% clay), and compared to those grown with a coconut and vermiculite substrate (50:50). The pots were irrigated with fertiliser-free process water from EAU and IAU production of *C. gariepinus* (“organo-hydroponics”) and compared to a hydroponic control with commercial fertiliser. The mineral content of the plants was tested twice by *Analysis I* (2020) to determine the elements and *Analysis II* (2023) to verify the values. The influence of substrates and aquaculture process water effluents on the plant growth performance is discussed and differences of wheat nutrient compositions are analysed.

## Materials and methods

### Experimental system design

**Fig. 1:**
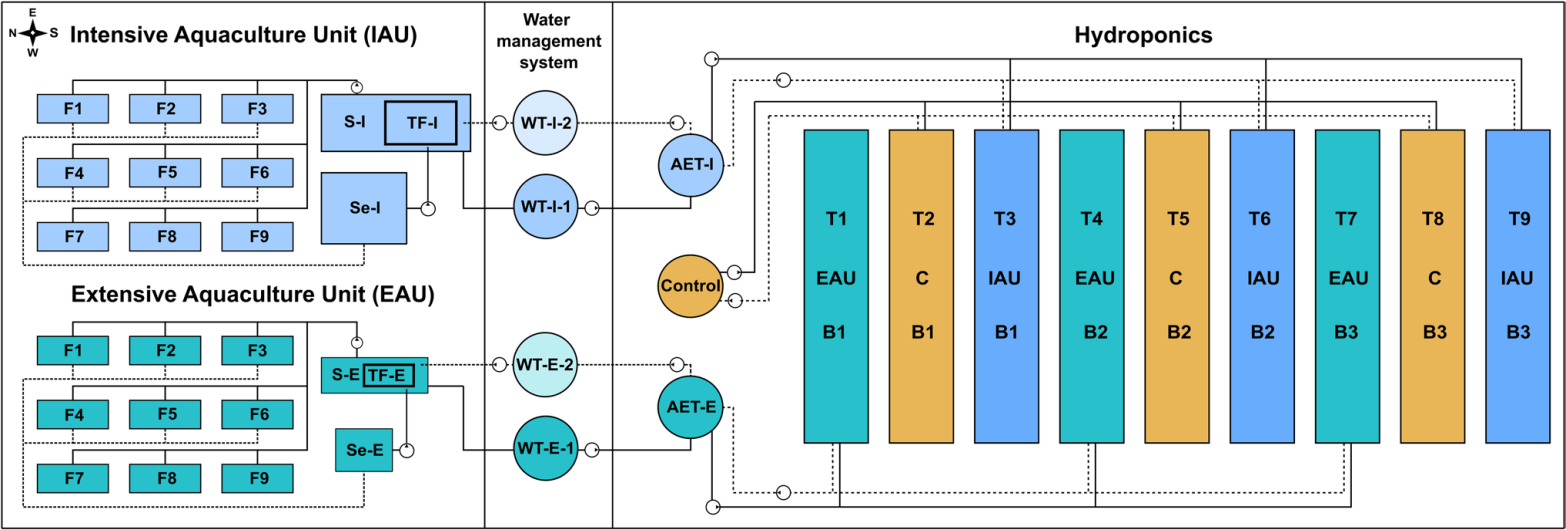
Schematic view of the FishGlassHouse operating units; inflows are shown with continuous lines and return flows with dotted lines, an intensive aquaculture unit (IAU), and an extensive aquaculture unit (EAU), associated flows through the water management system, and a hydroponics section with planting tables (T 1–9)

The experiment was conducted in the summer of 2020 at the FishGlassHouse (FGH) experimental and production facility, Faculty of Agricultural and Environmental Sciences, University of Rostock (UoR, Mecklenburg-Western Pomerania, Northern Germany; GPS: latitude: 54.075714, longitude: 12.096591). The FGH (total area 1200 m^2^) consists of six hydroponic cabins with a greenhouse area of 600 m^2^, laboratories (200 m^2^), a water management system (100 m^2^) and three aquaculture units (PAL Anlagenbau GmbH, Abtshagen, Germany), of which the extensive aquaculture unit (EAU, 100 m^2^; total water volume 13.9 m^3^) and the intensive aquaculture unit (IAU, 100 m^2^; total water volume 16.9 m^3^; Fig. 1) with three different stocking densities of *C. gariepinus* were used. Each aquaculture unit contained nine fish tanks of 1 m^3^ water volume (F 1–9) for staggered fish production (production of three fish age classes) arranged in triplicates (Rakocy et al. 2006), a solids separation unit (“sedimenter”, Se–E, Se–I), a trickling filter (TF–E, TF–I), and corresponding sumps (S–E, S–I; Fig. 1).

Aquaculture effluent water from the EAU and IAU was transferred through a water management system (area 100 m^2^) with water storage tanks (EAU: WT–E–1; IAU: WT–I–1, each approx. 1,500 L) to the hydroponic unit (100 m^2^; Fig. 1) which was built as a VENLO greenhouse (GTW Gewächshaustechnik Werder GmbH, Werder, Germany) with automatic climate control (Hempel und Rülcker, Gesellschaft für elektronische Klimaregelsysteme GmbH, Dresden, Germany). In the hydroponics, the process water was stored in associated aquaculture effluent tanks (EAU: AET–E; IAU: AET–I); in addition, the control group with fertilised tap water (processed from the nearby river “Warnow”) was stored in a separate nutrient tank (control), whereby each tank had a total volume of approx. 1000 L. Aquaculture effluent tanks and the fertiliser nutrient tank were connected to the hydroponics with a separate water recirculation system (Fackler Gewächshaustechnik, Munningen-Laub, Germany) that pumped the process water to nine ebb-and-flood tables (3.05 × 1.01 m/table, Otte Metallbau GmbH & Co. KG, Germany, slope: 1.68 × 0.89%, *n* = 3). All tanks were continuously aerated by an aeration stone using an air pump (Mistral 4000, Aqua Medic GmbH, Bissendorf, Germany). The tables were arranged in triplicates and three blocks (B 1–3) for the EAU (Tables: T/1,4,7/EAU, B 1–3), control (Tables: T/2,5,8/C, B 1–3), and IAU (Tables T/3,6,9/IAU, B 1–3) and contained the plant pots with *T. aestivum*. Three times a day (10:45 h, 16:45 h, 22:45 h) by using an automatic clock timer, the tables were flooded (repeated three times) in one direction (4 min flooding, 4 min outflow by gravity, 8 min total irrigation time), and then the water was pumped back to the aquaculture effluent (AET–E; AET–I) and fertiliser nutrient (control) tanks. Aquaculture effluents from AET–E and AET–I were pumped back to aquaculture units via water management system and associated tanks (WT–E–2, WT–I–2; Fig. 1) and exchanged every second day (excluding weekend) with fresh process water (via WT–E–1, WT–I–1)—three times (after first loading with process water on 20.07.2020) during the experiment (22.07.2020, 24.07.2020, 27.07.2020)—and pumped to hydroponics again. Nutrient solution of the control group was replaced with fresh water as needed and an electrical conductivity of 1600 µs/cm by adding the commercial fertiliser Universol^®^ Basis 4–19–35 + 4.1MgO + TE (ICL Specialty Fertilizers, ICL SF Germany & Austria) with an elemental composition of 4.0% NO_3_–N, 8.3% P, 29.0% K, 2.5% Mg, 6.2% S, 0.08% Mn, 0.02% B, 0.020% Cu, 0.002% Mo, and 0.020% Zn, as well as an oxide composition of 4.0% N–NO_3_, 19.0% P_2_O_5_, 35.0% K_2_O, 4.1% MgO, 15.5% SO_3_, 0.12% Fe (0.08% DTPA–chelate, 0.04% chelated by EDDHA), 0.08% Mn (EDTA–chelate), 0.02% B, 0.020% Cu (EDTA–chelate), 0.002% Mo, and 0.020% Zn (EDTA–chelate). The hydroponic tank of the control was equipped with an automated pH–controller (Bluelab Corporation Limited, New Zealand) for pH adjustment at 6.5; for increasing pH, a “pH + up” solution (28.2% potassium hydroxide) was used, and for decreasing pH, a “pH-bloom” solution (59% phosphoric acid; Advanced Hydroponics of Holland, The Netherlands) was used.

The experiment was conducted during a typical grow-out period of fish production in the FGH while the growth parameters of *C. gariepinus* were measured from 20.07.2020 to 21.08.2020 (without first day, 32 days), and the plant data were collected between 17.07.2020 and 29.07.2020 due to the rapid growth of wheatgrass.

### Fish production

*C. gariepinus* was obtained from a local fish farm (Fischzucht Abtshagen GmbH & Co, KG, Germany) and stocked into the aquaculture units of the FGH before the experiment for normal production. As initial data on 20.07.2020, the nine fish tanks each (Fig. 1) contained in *i*) EAU: the initial fish mass: 341.2 ± 175.9 g (10.9 ± 5.5 kg/m^3^), and fish number per tank: 32.2 ± 1.1, and *ii*) IAU: the initial fish mass: 351.9 ± 199.4 g (42.0 ± 23.8 kg/m^3^), and fish number per tank: 119.2 ± 0.7. Fish weights and biomasses were measured using scales (PCE-BS 300, PCE Deutschland GmbH, Meschede, Germany; SBS–PF–A150/20, Steinberg Systems, Zielona Góra, Poland).

Fish were fed with commercial feed Special Pro EF 4.5 mm from Alltech Coppens GmbH (The Netherlands) with crude protein 42%, crude lipids 13%, crude fibre 1.5%, crude ash 7.6%, phosphorus 1.08%, calcium 1.7%, sodium 0.3%, vitamin A of 10,000 IE/kg and vitamin D_3_ of 2305 IE/kg, iron 60 mg/kg, calcium 5 mg/kg, copper 5 mg/kg, manganese 20 mg/kg, and zinc 60 mg/kg. The feeding bins above the tanks were manually filled and electronically controlled to distribute the feed four times a day using a feeding computer from Autosoft (Automation & Software Günther Tausch GmbH, Germany). Feeding amount was based on practical feed conversion rates for *C. gariepinus* following a calculated protocol (PAL Anlagenbau GmbH, Abtshagen, Germany).

### Plant cultivation

Seeds of winter wheat (*T. aestivum*, weight: 0.0437 ± 0.0083 g, length: 0.6 ± 0.04 cm, width: 0.34 ± 0.02 cm, *N* = 33; Biolandhof Knauf, Warenvertrieb GbR, Bad Rodach, Germany) were germinated in six equivalents of Eschenfelder sprout jars (preserving jars, 1.7 L), half filled with wheat grains and filled up with tap water. Pre-germination of seeds (weight: 0.0722 ± 0.0147 g, length: 0.72 ± 0.05 cm, width: 0.43 ± 0.04 cm, *N* = 33) took place for 60 h from 17.07.2020 to 19.07.2020.

On 20.07.2020, the hydroponic tanks were initially filled with fertilised tap water (control) and process water from the aquaculture units (EAU, IAU). The plants (germinated seeds with radicles) were transferred into commercial garden pots with a substrate mixture of coconut fibre (Coco-Mix Biobizz^®^, Biobizz World Wide Organics, Bilbao, Bizkaia, Basque Country, Spain) and vermiculite (1–2 mm, Duengerexperte.de, Kipfenberg/Attenzell, Germany) with a ratio of 50:50. One seed of *T. aestivum* was sown in each small substrate pot (5 × 6 cm; total 135 pots; 15 pots per group and table) for individual plant growth parameters, and approx. 80 seeds were sown in large pots (11 × 11 cm; total 360 pots, 40 pots per group and table) for laboratory nutrient and vitamin analyses.

Another group of plants was additionally potted in small pots (5 × 6 cm; total 135 pots) of 100% conventional standard growing substrate, “Einheitserde”, with a composition of 80% white peat and 20% clay (EE–Typ 0: “Nullerde” after Fruhstorfer, Einheitserdewerke Patzer, Patzer Erden GmbH, Sinntal‒Altengronau, Germany).

**Fig. 2:**
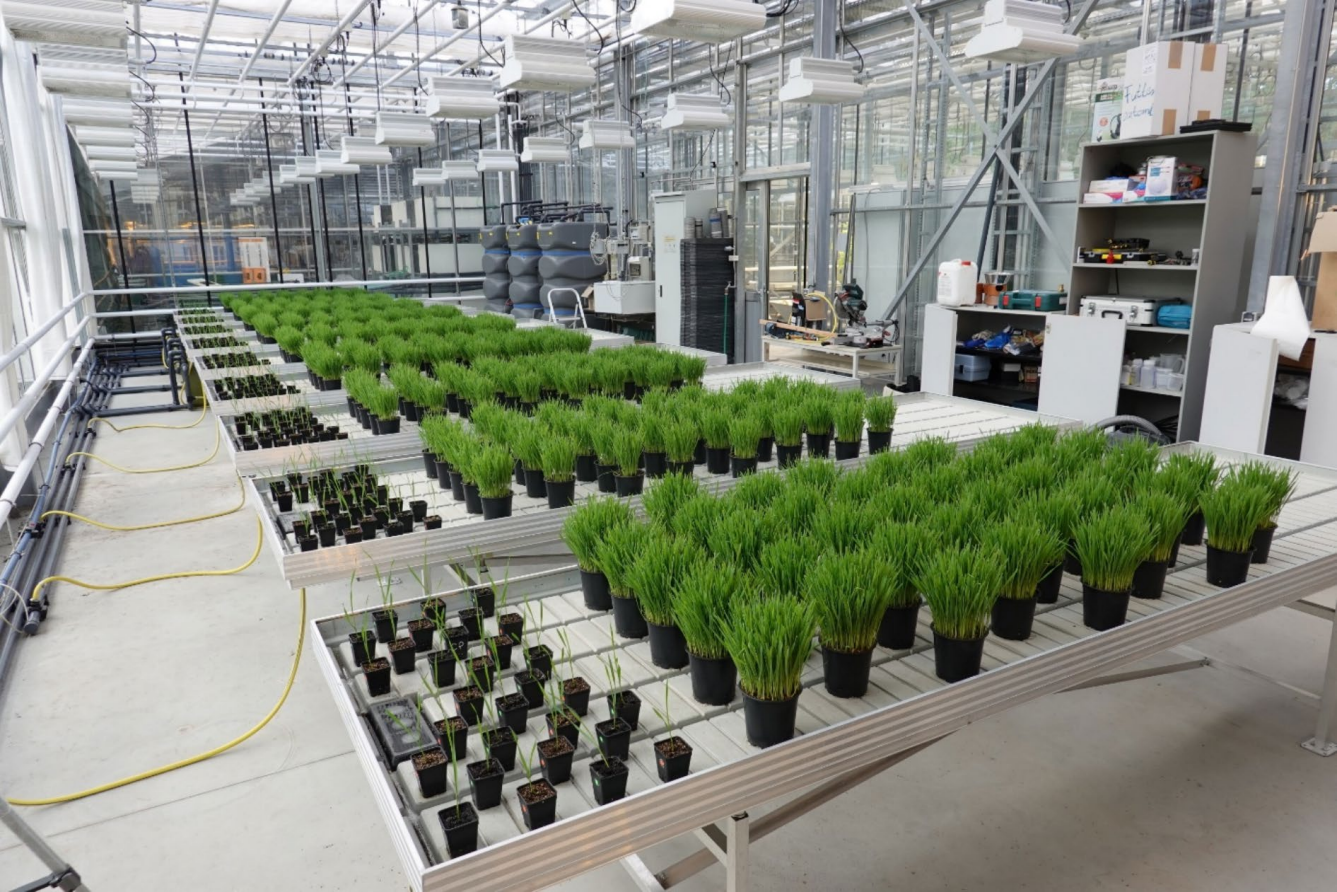
*Triticum aestivum* pots arranged in the hydroponic cabin on planting tables (FishGlassHouse)

The arrangement of the pots on the tables was diagonal for each size; the small pots were placed near the window in a westward direction, while the large pots were placed behind the small ones on the back part of the table (Fig. 2). On 22.7.2020, the first shoots appeared on the substrate surface. Growth parameters were recorded from the first observed green-coloured plant parts on 23.07.2020, and the experiment was terminated on 29.07.2020 (days after sowing (DAS) = 12 days; days after transplanting (DAT) = 9 days).

### Analysis of plant nutrient and growth parameters

Macro- and micronutrients of *T. aestivum*, with one sample from each planting table (*n* = 3 per group), were analysed by AGROLAB Agrar und Umwelt GmbH (Sarstedt, Germany) for *Analysis I*, as well as the pot substrates of “Einheitserde” and coconut and vermiculite substrate (50:50) by Raiffeisen–Laborservice (Ormont, Germany), and the plants’ crude nutrient and vitamin composition cultivated in coconut and vermiculite by SGS Germany GmbH (Hamburg, Germany). To validate the data, further wheatgrass plants were secondary cultivated (SC) after the experiment in triplicates also in coconut and vermiculite (50:50) substrate with irrigation of the IAU process water effluents (140 fish/tank), and with nearly the same growth period from 15.05.2023 to 26.05.2023 (11 days of growth, not including last day of data recording), and independent analyses of macro- and microelements were carried out by another laboratory (*Analysis II*, SGS INSTITUT FRESENIUS GmbH, Taunusstein, Germany).

Plant lengths were measured by hand ruler and the plant weights by scales (KB360–3N, d = 0.001 g, Kern & Sohn GmbH, Balingen-Frommern, Germany; ATX224, d = 0.1 mg, Shimadzu, Japan). The plant material was dried in an oven (UF750 plus, Memmert GmbH & Co. KG, Schwabach, Germany), the SPAD index was measured by SPAD-502PLUS (Konica Minolta. Inc., Marunouchi, Japan), and the colour parameters by Colorimeter PCE-CSM 2 (PCE Deutschland GmbH, Meschede, Germany).

### Physico-chemical parameters

Physical water parameters were taken each day (three replicates) at 14:00, from the hydroponic aquaculture process water and fertiliser control tanks, including temperature (°C), dissolved oxygen (DO, mg/L), oxygen saturation (%), conductivity (EC, µS/cm), pH, and redox potential (mV) with a HQ40D multimeter (Hach Lange GmbH, Düsseldorf, Germany). Air temperature (°C) and relative humidity (%) were recorded by the sensor system of the FishGlassHouse (Hempel und Rülcker, Gesellschaft für elektronische Klimaregelsysteme GmbH, Dresden, Germany).

Light parameters ware taken each day at 15:00 in the middle of the planting tables at a height of 10 cm (photosynthetic photon flux density PPFD, µmol/m^2^s) with a Lightscout–3415FSE Solar Electric Quantum Meter (Spectrum Technologies. Inc., USA), and light intensity with a LM–200 Lux meter (Eurolite, Steinigke Showtechnic GmbH, Waldbüttelbrunn, Germany).

The chemical water parameters were determined by taking samples from the hydroponic tanks and frozen at − 7 °C. Samples were later analysed with a Gallery™ Automated Photometric Analyzer (Thermo Fisher Scientific, Waltham, MA, USA) for ammonium (NH_4_^+^), nitrite (NO_2_^−^), nitrate (NO_3_^−^), phosphate (PO_4_^3−^), potassium (K^+^), magnesium (Mg^2+^), calcium (Ca^2+^), iron (Fe^2+^), and sulphate (SO_4_^2−^). The colorimetric hydrazine method (template: D08896_01^©^ 2015 Thermo Fisher Scientific Inc., Waltham, MA, USA) was used to analyse TON (total oxidized nitrogen) and nitrate by calculation (TON-nitrite).

### Mathematical and statistical analyses

Fish growth parameters were calculated by initial and final weight values of:

Feed conversion ratio (FCR; Boyd et al. 2007, 1):

1 $$\text{FCR}=\frac{\text{fish feed quantity }\left(\text{kg}\right)}{\text{weight gain }(\text{kg})}$$

Specific growth rate (SGR; Kaufmann 1981, 2):

2 $$SGR\, \left(\%\,{ d}^{-1}\right)=\frac{\text{ln}{W}_{t}-\text{ln}{W}_{0}}{t} \times 100$$

with *W*_*t*_ denotes final fish biomass (kg), *W*_*0*_ indicate initial fish biomass (kg), and *t* represents time in days; and absolute growth or weight gain (Schreck and Moyle 1990; 3):

3 $$\text{Weight gain }(\text{kg})=\text{final fish weight }\left(\text{kg}\right)-\text{ initial fish weight }(\text{kg})$$

Feed conversion ratio (FCR) and specific growth rate (SGR) were calculated by tank total fish biomasses with nine tanks per unit and three tanks per group (*n* = 3 × 3 = 9). Individual fish growth performance was calculated by randomized fish samples from each tank with three individuals per tank (*n* = 3 per tank, *n* = 9 per group).

The element and vitamin data were converted from fresh mass (f.m.) to dry mass (d.m.) using the following formula (modified after Hill labs 2023; 4):

4 $$E\, \&\, V (d.m.)=\frac{E\, \&\, V (f.m.)}{(\left(100 -M\right)/100)}$$

where *E* represents element, *V* stands for vitamin, and *M* for moisture (%) content.

All data were analysed using SPSS 29 statistical software package (IBM 2024) at a significance level of p ≤ 0.05. Two experimental groups were analysed by *t*-test if values were normally distributed and sample sizes were equal; otherwise, the Mann–Whitney test was used. Three experimental groups with normally distributed data and equal sample sizes were analysed by one-way ANOVA and a post hoc Tukey’s HSD test if variances were homogeneous, and by a Dunnett–T3 test at inhomogeneous variances. Values with different sample sizes and not normally distributed data were compared by Kruskal–Wallis ANOVA and Bonferroni correction.

## Results

### Fish production

Growth performance of *C. gariepinus* in the aquaculture units was not significantly different between initial and final individual fish weight, feed conversion ratio, specific growth, and mortality (Table 1). Individual fish weight gain was approx. 1.3-fold higher in EAU compared to IAU production, whereas biomass weight gain was nearly threefold significant greater in the IAU.

**Table 1:** Comparison of *C. gariepinus* growth performance parameters from extensive (EAU; fish number per tank: 32.2 ± 1.1) and intensive (IAU; fish number per tank: 119.2 ± 0.7) aquaculture units during 32 days of production; means (± SD), different letters indicate different groups (*p* < 0.05)

Parameters	EAU	IAU	*p*-value
Initial individual fish weight (g)	341.2 ± 175.9 ^a^	351.9 ± 199.4 ^a^	0.906
Final individual fish weight (g)	522.8 ± 187.2 ^a^	491.7 ± 184.3 ^a^	0.728
Individual fish weight gain (g)	181.6 ± 15.5 ^a^	139.9 ± 27.2 ^b^	0.001
Initial biomass stocking density (kg/m^3^)	10.9 ± 5.5 ^b^	42.0 ± 23.8 ^a^	0.004
Final biomass stocking density (kg/m^3^)	16.6 ± 5.6 ^b^	57.9 ± 21.7 ^a^	0.001
Biomass weight gain (kg/m^3^)	5.6 ± 0.4 ^b^	16.0 ± 3.6 ^a^	0.001
Total feed per tank (kg/m^3^)	6.6 ± 1.5 ^b^	24.0 ± 5.9 ^a^	0.001
FQ^1^	1.17 ± 0.27 ^a^	1.64 ± 0.78 ^a^	0.116
SGR (%/d)^1^	1.53 ± 0.62 ^a^	1.31 ± 0.82 ^a^	0.537
Mortality (%)^1^	1.05 ± 1.58 ^a^	1.11 ± 1.61 ^a^	0.730

^1^Calculated by tank biomasses

### Plant production

#### Hydroponic and greenhouse parameters

Physical water parameters such as dissolved oxygen, light intensity and PPFD in hydroponics were similar between aquaculture irrigation groups (EAU, IAU) and the control (C; Table 2). Temperature and pH were significantly higher in the IAU and the EAU than in the control. Throughout the experiment, the average air temperature was 22.7 ± 0.5 °C (min. 20.5 ± 0.2 °C; max. 26.7 ± 1.2 °C), and the relative humidity was 58.5 ± 6.0% (min. 39.6 ± 7.8%; max. 74.1 ± 3.3%; data received from FGH sensors).

**Table 2:** Comparison of physical (*n* = 30) and chemical (*n* = 30) water parameters of hydroponic aquaculture effluent tanks from intensive (IAU) and extensive (EAU) *C. gariepinus* production, and control group tank (C) with fertiliser, and light parameters (*n* = 21) above tables; different letters indicate different groups (*p* < 0.05)

Parameters	Control (C)	Extensive (EAU)	Intensive (IAU)	*p*-I^1^	*p*-II^1^	*p*-III^1^
O_2_ (mg/L)	8.7 ± 0.2^a^	8.6 ± 0.1^a^	8.6 ± 0.2^a^	0.344	0.344	0.344
O_2_ (%)	105.2 ± 1.1^b^	106.8 ± 1.0^a^	106.3 ± 1.1^a^	0.001	0.005	0.188
Temperature (°C)	24.9 ± 1.4^b^	25.7 ± 0.3^a^	25.8 ± 0.4^a^	0.001	0.001	1.000
pH	6.5 ± 0.2^b^	7.1 ± 0.3^a^	7.2 ± 0.1^a^	0.001	0.001	0.983
EC (µS/cm)	1629.4 ± 56.1^a^	717.0 ± 76.9^c^	961.4 ± 133.0^b^	0.001	0.001	0.001
RedOx (mV)	203.8 ± 23.9^a^	171.4 ± 12.3^b^	160.8 ± 17.5^b^	0.001	0.001	0.206
Light intensity (lx)	35,278.8 ± 33,269.8^a^	32,947.8 ± 30,772.7^a^	33,589.3 ± 32,972.9^a^	0.864	0.864	0.864
PPFD (µmol/m^2^s)	585.3 ± 464.4^a^	559.3 ± 447.4^a^	582.7 ± 464.5^a^	0.950	0.950	0.950
Ca^2+^ (mg/L)	78.28 ± 11.92^c^	130.24 ± 7.83^b^	203.99 ± 9.62^a^	0.001	0.001	0.001
Fe^2+^ (mg/L)	0.00 ± 0.01^c^	0.01 ± 0.01^b^	0.02 ± 0.01^a^	0.001	0.001	0.020
K^+^ (mg/L)	272.37 ± 14.58^a^	9.18 ± 3.05^c^	15.91 ± 3.41^b^	0.001	0.001	0.001
Mg^2+^ (mg/L)	71.69 ± 7.19^a^	26.10 ± 1.59^b^	27.76 ± 6.61^b^	0.001	0.001	0.052
NH_4_^+^ (mg/L)	0.39 ± 0.30^b^	0.57 ± 0.40^a,b^	0.65 ± 0.34^a^	0.095	0.004	0.837
NO_2_^−^ (mg/L)	0.02 ± 0.01^b^	0.33 ± 0.16^a^	0.37 ± 0.31^a^	0.001	0.001	1.000
NO_3_^−^ (mg/L)	396.09 ± 186.05^b^	252.28 ± 9.61^c^	655.50 ± 72.60^a^	0.002	0.001	0.001
PO_4_^3−^ (mg/L)	268.93 ± 23.26^a^	15.43 ± 4.70^c^	28.40 ± 6.07^b^	0.001	0.001	0.001
SO_4_^2−^ (mg/L)	341.99 ± 21.45^a^	135.21 ± 15.38^c^	157.16 ± 11.46^b^	0.001	0.001	0.006

^1^Significance (*p*) with: *p*‒I = control (C)/extensive (EAU); *p*‒II = control (C)/intensive (IAU); *p*‒III = extensive (EAU)/intensive (IAU)

Chemical water parameters reflected the proportion of increased feed input from the IAU and EAU aquaculture fish stocking densities. Conductivity (EC) showed a distinct trend: control > IAU > EAU. Similarly, P and K achieved the highest amounts in the control, followed by the IAU and the EAU, and Mg was lower and similar in both aquaponic groups. Ca and Fe reached the highest significant levels in the IAU, followed by the EAU and the control. NO_3_ showed the greatest significant values in the IAU, followed by the control and the EAU.

The subsequent cultivation (SC) of *T. aestivum* (*Analysis II*) irrigated with process water from the IAU showed little differences to the primary cultivation in IAU. EC was 2.3-fold higher (2232.42 ± 42.19 µS/cm), and pH was lower with 6.53 ± 0.10, whereas dissolved oxygen and RedOx were both little higher (9.55 ± 0.16 mg/L; 164.18 ± 36.44 mV), while temperature was lower (21.65 ± 1.17 °C).

#### Substrate composition

According to Table 3, nutrient parameters were not significantly different in soluble N fraction, Ca, and Zn. Furthermore, Einheitserde (E) showed in the fresh matter fraction significantly higher values of pH (E: 6.9 ± 0.0; C/V: 6.6 ± 0.1, *p* = 0.001), maximum methane yield (E: 83.7 ± 1.2 Nm^3^/t; C/V: 57.7 ± 1.5 Nm^3^/t, *p* = 0.046), and density (E: 0.4 ± 0.0 kg/L; C/V: 0.3 ± 0.0 kg/L, *p* = 0.037). Moreover, C/V substrate showed higher levels of dry matter (C/V: 52.0 ± 0.6%; E: 34.3 ± 0.5%, *p* = 0.001), and C:N ratio (C/V: 50.3 ± 7.0; E: 39.0 ± 1.7, *p* = 0.046).

**Table 3:** Comparison of basic macro- and micronutrient composition in the dry matter (d.m.) and fresh matter (f.m.) fraction between 100% Einheitserde (E) and coconut and vermiculite substrate (50:50; C/V); different letters indicate different groups (*p* < 0.05)

Substrates	E	C/V	*p*-value	E	C/V	*p*-value
Parameters	Dry matter (d.m.)			Fresh matter (f.m.)		
Organic matter (g/kg)	660.8 ± 4.8 ^a^	301.6 ± 11.8 ^b^	0.001	226.5 ± 3.3 ^a^	156.7 ± 4.4 ^b^	0.001
Total N (g/kg)	10.6 ± 0.4 ^a^	3.7 ± 0.3 ^b^	0.046	1.6 ± 0.1 ^a^	0.5 ± 0.0 ^b^	0.043
Organic N content (g/kg)	10.4 ± 0.4 ^a^	3.6 ± 0.3 ^b^	0.046	1.6 ± 0.1 ^a^	0.4 ± 0.1 ^b^	0.043
Soluble N fraction (g/kg)	0.1 ± 0.0 ^a^	0.1 ± 0.1 ^a^	0.317	0.0 ± 0.0 ^a^	0.0 ± 0.0 ^a^	0.114
Phosphate (P_2_O_5_) (g/kg)	0.7 ± 0.1 ^b^	12.6 ± 2.0 ^a^	0.046	0.1 ± 0.0 ^b^	1.6 ± 0.3 ^a^	0.037
Potassium (K_2_O) (g/kg)	0.7 ± 0.1 ^b^	4.5 ± 0.3 ^a^	0.046	0.1 ± 0.0 ^b^	0.6 ± 0.0 ^a^	0.046
Magnesium (MgO) (g/kg)	2.1 ± 0.1 ^b^	29.3 ± 0.1 ^a^	0.001	0.3 ± 0.0 ^b^	3.8 ± 0.0 ^a^	0.034
Calcium (CaO) (g/kg)	31.9 ± 0.5 ^a^	31.3 ± 5.1 ^a^	0.849	4.8 ± 0.2 ^a^	4.0 ± 0.7 ^a^	0.099
Sodium (Na_2_O) (g/kg)	0.4 ± 0.1 ^b^	0.7 ± 0.0 ^a^	0.034	0.1 ± 0.00 ^b^	0.1 ± 0.0 ^a^	0.034
Sulphur (S) (g/kg)	2.3 ± 0.2 ^a^	0.6 ± 0.1 ^b^	0.046	0.3 ± 0.0 ^a^	0.1 ± 0.0 ^b^	0.001
Boron (B) (mg/kg)	1.7 ± 0.2 ^b^	3.5 ± 0.2 ^a^	0.001	0.3 ± 0.0 ^b^	0.4 ± 0.0 ^a^	0.001
Cobalt (Co) (mg/kg)	0.7 ± 0.1 ^b^	4.1 ± 0.3 ^a^	0.046	0.1 ± 0.0 ^b^	0.5 ± 0.0 ^a^	0.046
Iron (Fe) (mg/kg)	4618.5 ± 207.9 ^b^	5576.5 ± 379.2 ^a^	0.019	697.7 ± 27.3 ^a^	717.1 ± 44.9 ^a^	0.558
Copper (Cu) (mg/kg)	1.8 ± 0.1 ^b^	94.9 ± 2.1 ^a^	0.001	0.3 ± 0.0 ^b^	12.2 ± 0.3 ^a^	0.046
Manganese (Mn) (mg/kg)	87.3 ± 0.7 ^b^	97.3 ± 2.6 ^a^	0.003	13.2 ± 0.4 ^a^	12.5 ± 0.4 ^a^	0.128
Zinc (Zn) (mg/kg)	18.6 ± 3.9 ^a^	31.0 ± 18.3 ^a^	0.316	2.8 ± 0.6 ^a^	4.0 ± 2.4 ^a^	0.439

#### Plant growth

Considering Table 4 and plant cultivation in Einheitserde (100%), shoot length reached the highest value in the IAU. The highest value of root length was attained in the control, which was significantly higher than the EAU, but comparable to the IAU. Shoot wet weight was significantly higher in the IAU than the EAU but had no significant difference from the control. The growth parameters such as root/shoot dry weight ratio, the SPAD, and the luminance values, were not significantly different among the groups.

**Table 4:** Comparison of *T. aestivum* growth parameters cultured in Einheitserde (100%) and irrigated with aquaculture effluents from extensive (EAU) and intensive (IAU) *C. gariepinus* production and a control (C) with commercial fertiliser, 9 days after transplanting (DAT) and 12 days after sowing (DAS); different letters indicate different groups (*p* < 0.05)

Parameters	Control (C)	Extensive (EAU)	Intensive (IAU)	*p*-I^1^	*p*-II^1^	*p*-III^1^
Plant height (cm)	35.8 ± 4.4^a,b^	35.0 ± 4.2^b^	37.2 ± 3.2^a^	0.445	0.398	0.009
Shoot length (cm)	21.7 ± 3.0^b^	22.1 ± 3.1^b^	23.8 ± 2.2^a^	1.000	0.001	0.006
Root length (cm)	14.4 ± 2.2^a^	13.1 ± 2.0^b^	13.6 ± 2.0^a,b^	0.022	0.288	0.913
Plant wet weight (mg)	502.5 ± 141.4^a^	482.0 ± 149.8^a^	537.9 ± 122.2^a^	0.182	0.182	0.182
Shoot wet weight (mg)	354.5 ± 96.7^a,b^	326.8 ± 82.0^b^	380.8 ± 77.0^a^	0.174	0.830	0.008
Root wet weight (mg)	139.0 ± 52.6^a^	145.0 ± 72.3^a^	148.2 ± 55.0^a^	0.820	0.820	0.820
Shoot dry weight (mg)	39.2 ± 9.9^a^	37.1 ± 10.1^a^	40.8 ± 9.5^a^	0.196	0.196	0.196
Root dry weight (mg)	14.6 ± 5.3^a^	14.9 ± 5.7^a^	14.1 ± 4.1^a^	0.870	0.870	0.870
Root/shoot length ratio (cm)	0.67 ± 0.13^a^	0.61 ± 0.13^b^	0.57 ± 0.09^b^	0.007	0.001	1.000
Root/shoot dry weight ratio (mg)	0.37 ± 0.12^a^	0.40 ± 0.12^a^	0.35 ± 0.07^a^	0.175	0.175	0.175
SPAD (%)	33.3 ± 3.0^a^	34.4 ± 3.3^a^	34.3 ± 2.3^a^	0.133	0.133	0.133
Luminance (L*)	29.7 ± 3.4^a^	30.3 ± 4.5^a^	29.5 ± 4.4^a^	0.610	0.610	0.610
Red/green (a*)	− 8.3 ± 3.1^a^	− 8.1 ± 3.6^a^	− 9.7 ± 3.4^a^	0.063	0.063	0.063
Yellow/blue (b*)	19.1 ± 2.1^a^	19.0 ± 2.3^a^	19.3 ± 2.5^a^	0.646	0.646	0.646

^1^Significance (*p*) with: *p*‒I = control (C)/extensive (EAU); *p*‒II = control (C)/intensive (IAU); *p*‒III = extensive (EAU)/intensive (IAU)

The growth performance of *T. aestivum*, cultured in the coconut and vermiculite substrate (50:50), was significantly better with the IAU effluents (Table 5). The highest values were found in plant height, shoot length, plant wet weight, shoot wet weight, and shoot dry weight. SPAD obtained the highest value in the IAU and the EAU, and the control was not significantly different from the EAU. Parameters of luminance (L*), red/green (a*), and yellow/blue (b*) were not significantly different among experimental groups.

**Table 5:** Comparison of *T. aestivum* growth parameters cultured in the coconut and vermiculite substrate (50:50) and irrigated with aquaculture effluents from extensive (EAU) and intensive (IAU) *C. gariepinus* production and a control (C) with commercial fertiliser, 9 days after transplanting (DAT) and 12 days after sowing (DAS); different letters indicate different groups (*p* < 0.05)

Parameters	Control (C)	Extensive (EAU)	Intensive (IAU)	*p*-I^1^	*p*-II^1^	*p*-III^1^
Plant height (cm)	33.9 ± 4.3^b^	34.7 ± 3.7^b^	36.5 ± 3.8^a^	1.000	0.005	0.043
Shoot length (cm)	20.3 ± 2.8^b^	21.4 ± 2.7^b^	22.7 ± 2.4^a^	0.147	0.001	0.022
Root length (cm)	13.7 ± 2.8^a^	13.4 ± 2.2^a^	13.9 ± 2.6^a^	0.821	0.821	0.821
Plant wet weight (mg)	443.3 ± 142.9^b^	471.5 ± 135.3^b^	550.9 ± 115.5^a^	0.575	0.001	0.015
Shoot wet weight (mg)	291.4 ± 84.0^b^	313.6 ± 81.9^b^	362.3 ± 68.1^a^	0.383	0.001	0.012
Root wet weight (mg)	142.2 ± 63.8^b^	148.4 ± 65.9^a,b^	175.4 ± 49.8^a^	0.879	0.029	0.094
Shoot dry weight (mg)	33.4 ± 9.9^b^	33.9 ± 10.2^b^	39.6 ± 7.6^a^	0.968	0.006	0.012
Root dry weight (mg)	13.1 ± 5.0^a^	14.3 ± 4.7^a^	15.2 ± 3.6^a^	0.390	0.071	0.636
Root/shoot length ratio (cm)	0.69 ± 0.16^a^	0.63 ± 0.13^a^	0.62 ± 0.12^a^	0.072	0.072	0.072
Root/shoot dry weight ratio (mg)	0.39 ± 0.10^a,b^	0.43 ± 0.10^a^	0.38 ± 0.07^b^	0.194	0.985	0.043
SPAD (%)	30.9 ± 3.3^b^	31.7 ± 4.3^a,b^	32.9 ± 2.0^a^	0.197	0.007	0.689
Luminance (L*)	31.1 ± 3.8^a^	30.1 ± 4.8^a^	29.8 ± 4.1^a^	0.585	0.585	0.585
Red/green (a*)	− 8.9 ± 2.8^a^	− 9.8 ± 3.5^a^	− 9.9 ± 3.6^a^	0.336	0.336	0.336
Yellow/blue (b*)	20.1 ± 2.2^a^	19.9 ± 2.8^a^	20.1 ± 2.5^a^	0.923	0.923	0.923

^1^Significance (*p*) with: *p*‒I = control (C)/extensive (EAU); *p*‒II = control (C)/intensive (IAU); *p*‒III = extensive (EAU)/intensive (IAU)

#### Nutrient and vitamin composition of primary *T. aestivum* cultivation

 As shown in Table 6, the crude nutrient composition (fresh matter: f.m., *Analysis I*) of *T. aestivum* (plant structure with shoot axis and leaves) cultured in a coconut and vermiculite substrate (50:50) showed no differences in protein, lipid, ash, or fibre between the two irrigation groups of aquaculture effluents (EAU, IAU) and the control. Considering minerals nutrients, there were no significant differences in Mg, S, Sodium (Na), Cu, Fe, Zn, and Mo among all the groups. Elements like P, K, B, and Mn reached the highest amounts in the control and were not significantly different between the EAU and the IAU. Significantly higher levels of total N and Ca were attained in the IAU, which were comparable to the EAU. Vitamin content was not significantly different among groups, except for vitamin B_6_, which was significantly higher in the IAU and comparable between the EAU and the control.

**Table 6:** Comparison of *T. aestivum* above-ground samples (shoots and leaves, *Analysis I*) in crude nutrient composition (f.m. basis), macro- and micronutrients and specific vitamins (d.m. basis), cultured in coconut and vermiculite substrate (50:50) irrigated with aquaculture effluents from extensive (EAU) and intensive (IAU) *C. gariepinus* production and a control (C) with commercial fertiliser, 9 days after transplanting (DAT) and 12 days after sowing (DAS); data with < were below the detection threshold; different letters indicate different groups (*p* < 0.05)

Parameters	Control (C)	Extensive (EAU)	Intensive (IAU)	*p*-I^1^	*p*-II^1^	*p*-III^1^
Moisture (%)	88.81 ± 1.51^a^	88.75 ± 0.49^a^	89.46 ± 0.59^a^	0.997	0.711	0.668
Crude protein (%)	2.87 ± 0.38^a^	2.90 ± 0.10^a^	2.93 ± 0.12^a^	0.698	0.698	0.698
Crude lipid (%)	0.70 ± 0.00^a^	0.73 ± 0.06^a^	0.63 ± 0.06^a^	0.110	0.110	0.110
Ash (%)	1.99 ± 0.26^a^	1.69 ± 0.04^a^	1.60 ± 0.08^a^	0.342	0.229	0.450
Crude fibre (%)	2.47 ± 0.35^a^	2.63 ± 0.23^a^	2.63 ± 0.42^a^	0.775	0.775	0.775
Total-nitrogen (g/kg)	41.36 ± 0.63^b^	41.49 ± 1.07^a,b^	43.52 ± 0.76^a^	0.982	0.045	0.057
Total phosphorus (g/kg)	12.67 ± 0.30^a^	6.11 ± 0.20^b^	6.56 ± 0.15^b^	0.001	0.001	0.107
Potassium (g/kg)	76.52 ± 3.05^a^	68.63 ± 2.05^b^	66.18 ± 2.62^b^	0.023	0.007	0.520
Magnesium (g/kg)	2.26 ± 0.06^a^	2.19 ± 0.07^a^	2.25 ± 0.04^a^	0.376	0.994	0.422
Calcium (g/kg)	1.77 ± 0.09^b^	1.96 ± 0.05^a,b^	2.14 ± 0.14^a^	0.123	0.010	0.171
Sulphur (g/kg)	5.79 ± 0.40^a^	5.26 ± 0.09^a^	5.15 ± 0.17^a^	0.061	0.061	0.061
Sodium (g/kg)	0.54 ± 0.08^a^	0.35 ± 0.07^a^	0.29 ± 0.04^a^	0.051	0.051	0.051
Boron (mg/kg)	6.77 ± 0.19^a^	4.44 ± 0.05^b^	3.81 ± 0.59^b^	0.003	0.018	0.396
Copper (mg/kg)	12.41 ± 0.35^a^	12.22 ± 0.14^a^	12.21 ± 0.66^a^	0.861	0.850	1.000
Iron (mg/kg)	78.27 ± 4.94^a^	83.80 ± 7.09^a^	83.35 ± 6.06^a^	0.542	0.592	0.995
Manganese (mg/kg)	59.09 ± 1.02^a^	47.71 ± 1.36^b^	50.54 ± 2.79^b^	0.001	0.004	0.237
Zinc (mg/kg)	32.18 ± 0.64^a^	33.00 ± 0.75^a^	33.58 ± 0.52^a^	0.328	0.082	0.546
Molybdenum (mg/kg)	1.42 ± 0.06^a^	1.28 ± 0.05^a^	1.27 ± 0.02^a^	0.061	0.061	0.061
Pyridoxal (mg/100 g)	< 0.010	< 0.010	< 0.010	-	-	-
Pyridoxamine (mg/100 g)	0.29 ± 0.05^a^	0.33 ± 0.03^a^	0.38 ± 0.06^a^	0.586	0.136	0.473
Pyridoxine (mg/100 g)	0.25 ± 0.07^a^	0.25 ± 0.08^a^	0.40 ± 0.05^a^	0.066	0.066	0.066
Vitamin B_6_ (mg/100 g)	0.54 ± 0.10^b^	0.58 ± 0.06^b^	0.78 ± 0.07^a^	0.802	0.021	0.044
Vitamin B_7_ (Biotin) (µg/100 g)	< 5.00	< 5.00	< 5.00	-	-	-
Folic acid B_9_ (µg/100 g)	1203.34 ± 881.03^a^	1057.29 ± 552.29^a^	1385.38 ± 848.57^a^	0.971	0.956	0.865
Vitamin B_12_ (µg/100 g)	3.73 ± 2.24^a^	3.86 ± 1.35^a^	1.66 ± 0.15^a^	0.066	0.066	0.066
Vitamin E (mg/100 g)	2.60 ± 0.42^a^	3.14 ± 1.04^a^	2.67 ± 0.84^a^	0.710	0.995	0.766
Alpha—Tocopherol (mg/100 g)	2.60 ± 0.42^a^	3.13 ± 1.04^a^	2.66 ± 0.83^a^	0.716	0.996	0.764
Gamma—Tocopherol (mg/100 g)	0.00 ± 0.00^a^	0.03 ± 0.05^a^	0.03 ± 0.06^a^	0.558	0.558	0.558

^1^ Significance (*p*) with: *p*‒I = control (C)/extensive (EAU); *p*‒II = control (C)/intensive (IAU); *p*‒III = extensive (EAU)/intensive (IAU)

#### Nutrient composition of *T. aestivum* subsequent cultivation

The secondary cultivation (SC) of *T. aestivum* (*Analysis II*), irrigated only with process water from the IAU achieved comparable results to the primary cultivation of wheat in the IAU combined with C/V substrate (50:50; Table 6). No significant differences were found in moisture (89.42 ± 0.32%, *p* = 0.936), crude protein content (3.20 ± 0.26%, *p* = 0.200), crude lipid (0.60 ± 0.10%, *p* = 0.700), and fibre content (2.47 ± 0.40%, *p* = 0.645), whereas ash amount was significantly higher (1.96 ± 0.05%, *p* = 0.003). Most of the elements in the dry matter fraction of the leaves were comparable, such as P (6.26 ± 0.28 g/kg, *p* = 0.171), K (67.64 ± 3.71 g/kg, *p* = 0.606), Mg (2.68 ± 0.39 g/kg, *p* = 0.134), Cu (13.33 ± 1.53 mg/kg, *p* = 0.309), Fe (94.00 ± 43.49 mg/kg, *p* = 0.714), and Mn (44.67 ± 7.64 mg/kg, *p* = 0.279). In *Analysis II*, higher levels were found in Ca (3.64 ± 0.40 g/kg, *p* = 0.003), Na (0.54 ± 0.06 g/kg, *p* = 0.004) and Zn (39.00 ± 1.73 mg/kg, *p* = 0.046); whereas lower values were found in S (3.61 ± 0.29 g/kg, *p* = 0.046) and Mo (1.00 ± 0.00 mg/kg, *p* = 0.001).

#### Wheatgrass growth in different substrates

**Fig. 3:**
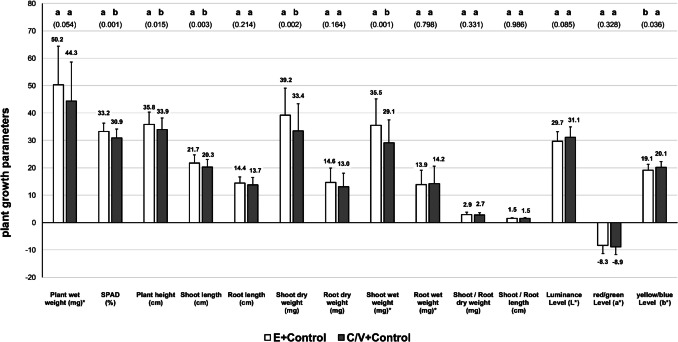
Comparison of *T. aestivum* plant growth parameters cultured in Einheitserde substrate (E, 100%) and coconut and vermiculite substrate (C/V, 50:50) irrigated with commercial fertiliser (control), 9 days after transplanting (DAT) and 12 days after sowing (DAS); different letters indicate different groups (*p* < 0.05), *p*-values in brackets below; values marked with ‘*’ were divided by 10 (×/10)

Plants cultivated in Einheitserde substrate (E) of control groups showed improved parameters for SPAD values, plant height, shoot length, as well as shoot dry weight and shoot wet weight (Fig. 3).

**Fig. 4:**
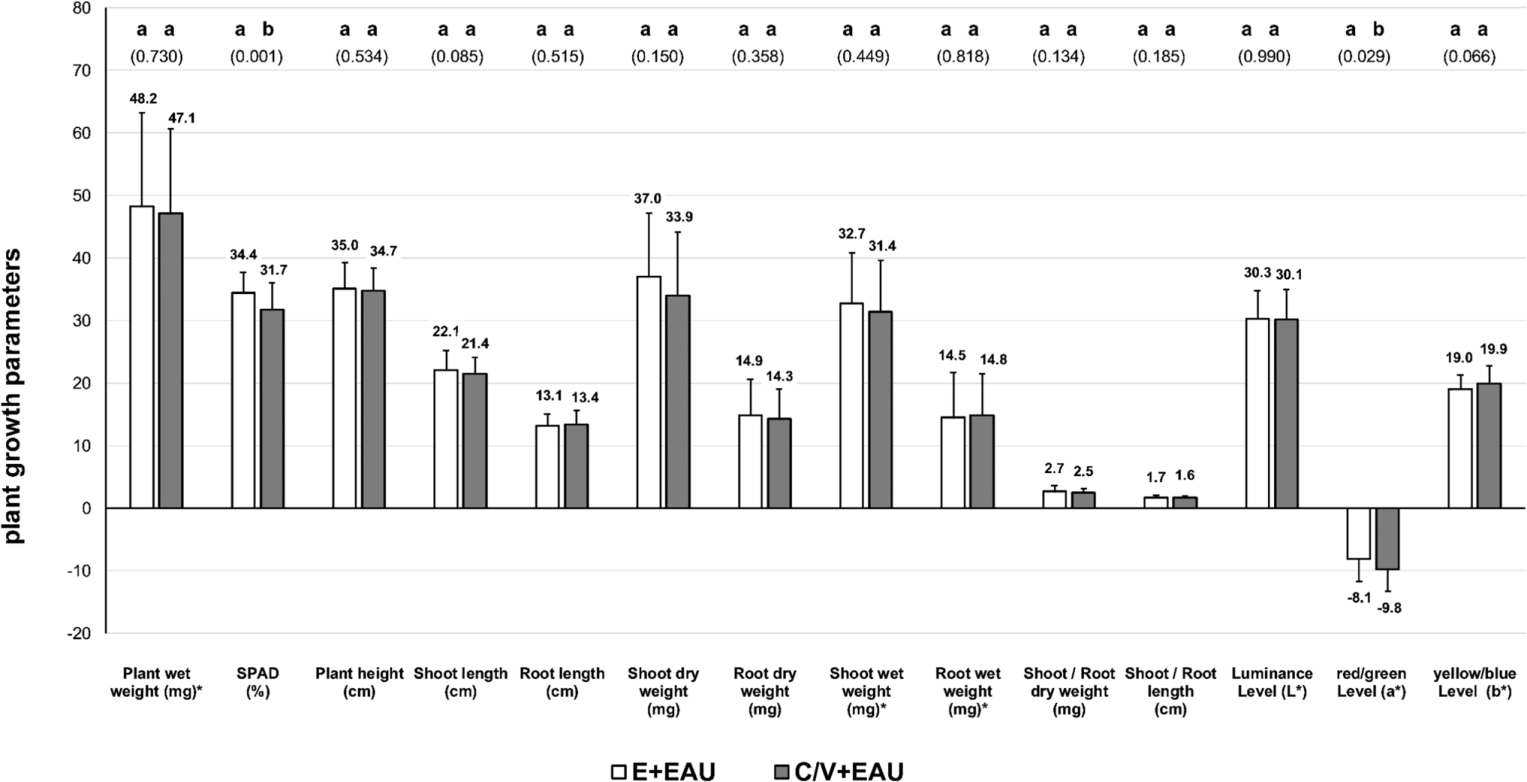
Comparison of *T. aestivum* plant growth parameters cultured in Einheitserde substrate (E, 100%) and coconut and vermiculite substrate (C/V, 50:50) irrigated with extensive aquaculture process water (EAU), 9 days after transplanting (DAT) and 12 days after sowing (DAS); different letters indicate different groups (*p* < 0.05), *p*-values in brackets below; values marked with ‘*’ were divided by 10 (×/10)

In plants irrigated with aquaponic process water from the EAU, only SPAD and red/green levels were significantly higher in the Einheitserde substrate, while all other parameters were comparable between both substrate groups (Fig. 4).

**Fig. 5:**
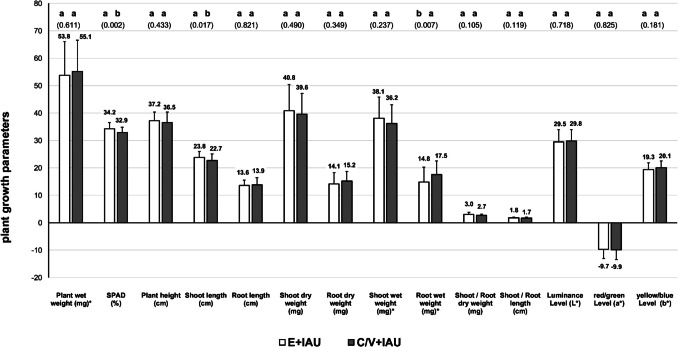
Comparison of *T. aestivum* plant growth parameters cultured in Einheitserde substrate (E, 100%) and coconut and vermiculite substrate (C/V, 50:50) irrigated with intensive aquaculture process water (IAU), 9 days after transplanting (DAT) and 12 days after sowing (DAS); different letters indicate different groups (*p* < 0.05), *p*-values in brackets below; values marked with ‘*’ were divided by 10 (×/10)

Wheatgrass irrigated with process water from the IAU showed significantly higher SPAD values and shoot length in Einheitserde substrate, whereas in the C/V substrate, root wet weight was significantly higher (Fig. 5).

**Fig. 6:**
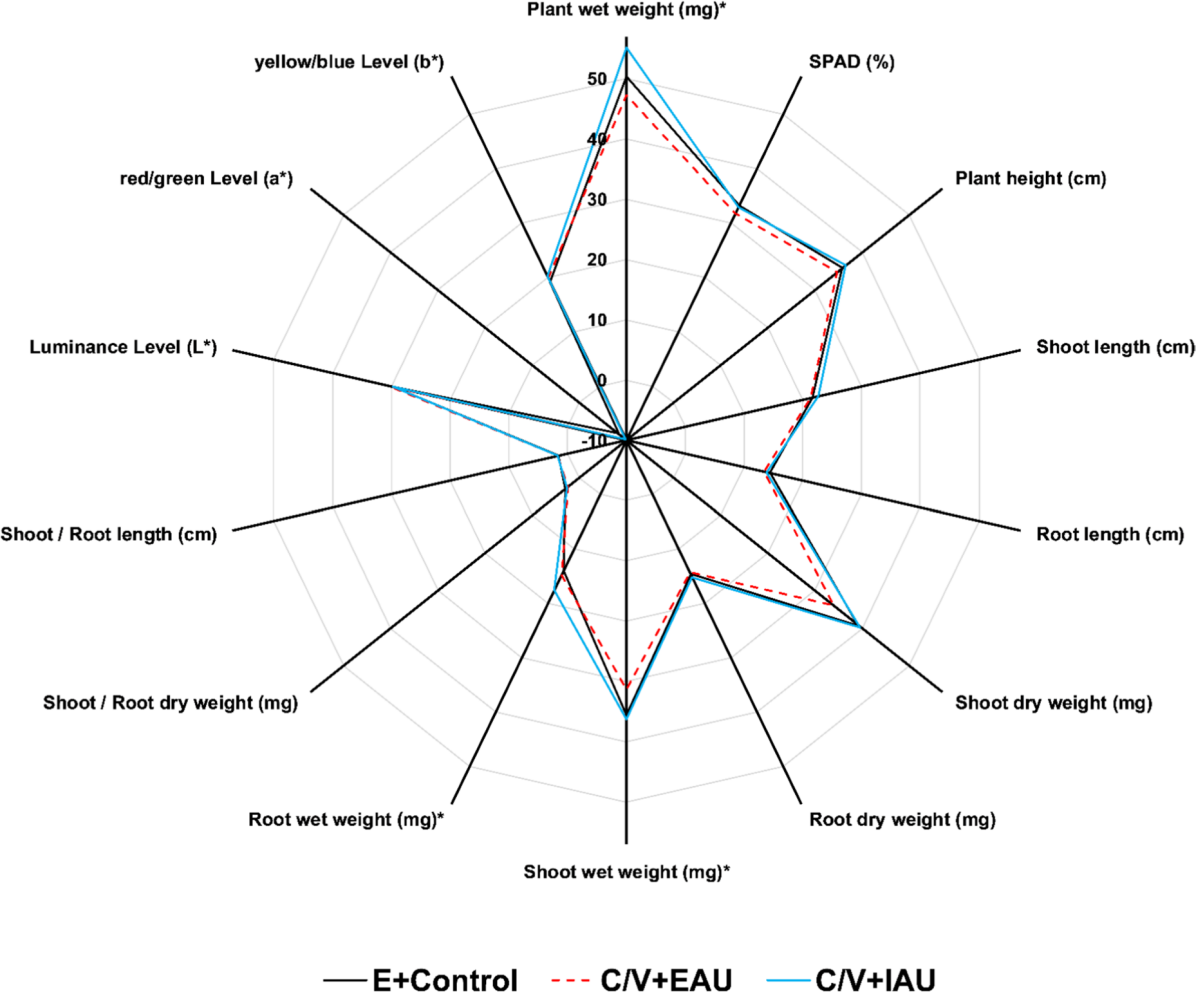
Comparison of *T. aestivum* plant growth parameters cultured in Einheitserde substrate (E, 100%) irrigated with commercial fertiliser (E + control), coconut and vermiculite substrate (C/V, 50:50) irrigated with extensive aquaculture process water (C/V + EAU), and coconut and vermiculite substrate (C/V, 50:50) irrigated with intensive aquaculture process water (C/V + IAU), 9 days after transplanting (DAT) and 12 days after sowing (DAS); values marked with ‘*’ were divided by 10 (×/10)

The comparison of plant growth in Einheitserde irrigated with fertiliser (E + control) in relation to aquaponic groups cultivated in the C/V substrate showed generally higher growth parameters in the IAU, of: plant wet weight, plant height, shoot length, shoot dry weight, root dry weight, shoot wet weight, root wet weight, shoot/root length, luminance level (L*), and yellow/blue level (b*, 10 of 14; Fig. 6). In contrast, plants grown in the C/V + EAU substrate showed better values compared to the E + control group only for: root wet weight, shoot/root length, luminance level (L*), and yellow/blue level (b*, 4 of 14; Fig. 6).

## Discussion

### Fish production

Growth performance of *C. gariepinus* was generally good, and no signs of diseases or deficiencies were observed. Fish growth parameters were not significantly different in feed conversion or specific growth (Table 1). However, Mota et al. (2015) reported better feed conversion of 0.97 at comparable age classes from 254.7 g to 503.6 g in a high accumulation recirculating aquaculture system (RAS) with large amount of suspended particulate matter (load of feed and bacteria); and an FCR of 0.87 (fish initial weight: 251.7 g; final weight: 549.7 g) in a low accumulation RAS which were not significantly different. Little younger *C. gariepinus* with an individual growth from 189.1 g to 456.2 g (after 29 days) showed also greater feed conversion from 0.90 to 0.99 by feeding different experimental basic diets with maize, wheat, barley, and rye (Leenhouwers et al. 2007). Furthermore, van de Nieuwegiessen et al. (2009) reported also better FCR of 0.81 in older *C. gariepinus* (final body weight: 1446.3 g) held under extensive stocking conditions (67 fish/m^3^) with specific growth of 1.27%/d which was slightly lower than in the present study. Comparable but slightly better FCR’s and SGR’s were reported by Palm et al. (2018) in the FGH over a longer growing period of 204 days with similar values of specific growth in extensive stocking with 1.77%/d (FCR: 0.87) and intensive with 1.80%/d (FCR: 0.97) with a fish growth of 40 g to 1492.0 g in the EAU and up to 1560.9 g in the IAU. Thus, fish growth was within known values for this species and showed no negative influence of stocking densities. The slightly poorer feed conversion may have been influenced by the shorter growing time (32 days) and the protein content of the feed, which was partly lower.

### Plant growth

#### Environmental culture parameters

In hydroponics, air temperature was within the optimal range of 18–24 °C (Kaur et al. 2022), and water temperature was slightly higher (≈ 2 °C) than 23 °C reported by Mackowiak et al. (1989) for cultivation in an NFT hydroponic solution. However, no heat stress occurred in the experiment, and in wheat, it can result in reduced growth, membrane damage, reduced enzyme activity, synthesis of stress-related proteins (heat shock proteins) and electrolyte loss (Heyne 1987; Kumar and Rai 2014). The optimal temperature range for wheat is narrow, and an increase one degree above 17 to 24 °C can, for example, reduce grain weight by four percent. This effect is more pronounced later during the pollination and milky-dough stages and less during the younger vegetative phase, as in this study (Kumar and Rai 2014). Furthermore, Thies et al. (2024) found that temperature has a greater influence on growth than light energy for photosynthesis. The threshold at which photosynthesis decreases was suspected to occur between 17.5 and 22.5 °C, which was slightly exceeded in the study by air temperature, but due to homogeneous experimental conditions, the growth of the different wheat grass groups was not affected. In contrast, water temperatures were slightly elevated in the aquaponic groups compared to the control (Table 2), influenced by higher production temperatures in the EAU and IAU for *C. gariepinus* (approx. 28 °C). A growth-promoting effect cannot be excluded; however, EAU exhibited reduced shoot lengths and dry weight compared to IAU (Tables 4 and 5), suggesting that the influence of nutrient solutions on growth was more significant than water temperatures.

Nutrient solutions used in the experiment had a pH value close to 7.0, which has been described as ideal for *T. aestivum* in hydroponics (Bhuyan et al. 2019). Eight-day-old wheat showed reduced plant height, biomass, chlorophyll content and oxidative damage at the extreme acidic pH of 4.0 and alkaline pH of 8.5 (Bhuyan et al. 2019). Furthermore, wheat appears to be relatively tolerant to acidic pH values of 5.5, as morphophysiological characteristics such as plant height improved significantly compared to the extremely acidic pH value of 4.4 (Bhuyan et al. 2019). In the study, pH values in the aquaponic groups were very slightly increased above 7 and little reduced by 0.5 in the control. Generally, most plants prefer a pH between 6.0 and 7.0 for optimal nutrient uptake (Junge et al. 2020). Wheat, however, shows reduced growth at a lower pH of 6.3–6.5 when cultivated in a modified Hoagland solution (8–10 days) at relatively comparable values of humidity at 70%, constant 21 °C air temperature, but a continuous 24 h photoperiod (Dong et al. 2014). The study showed that the shoot lengths between control and EAU in both substrates were not significantly different (Tables 4 and 5), suggesting only a small influence of the lower pH in control.

Conductivity was significantly different between groups with the highest value in the control (C > IAU > EAU; Table 2). Surprisingly, plants in the IAU showed the highest shoot length compared to control (Tables 4 and 5) although the EC value in IAU was reduced by 41% (Table 2). The same was reported by Xu et al. (2022) with a higher shoot dry mass of 97.10 mg in the IAU (1665.85 µS/cm) compared to 85.45 mg in the control (1837.54 µS/cm) in a similar and longer experiment duration of 6 days. The reason is unclear, but since conductivity is a relatively variable value due to different ion contents and charges, individual substances are decisive (Langenfeld et al. 2022). Apparently, the type of ions differed between the fertiliser group and the process water from the IAU, and was more strongly influenced by nitrogen-derived ions (nitrate) in the IAU (Table 2). Stress from NaCl can be excluded due to uniform content in the C/V substrate (Table 3) and since reduced leaf growth was not observed (Munns 2002). The influence of nitrogen (ammonium, nitrate; Table 2) appears to have been crucial for the better plant growth in the IAU. Nitrogen deficiency was also reported by Page and Feller (2013) in spring wheat with lower plant heights of 15–20 cm after 9 days of cultivation in an NFT hydroponic system and a 19% higher EC of 1.2 dS/m compared to IAU. To avoid negative effects on growth, such as premature senescence or delays (cell ageing process), and the accumulation of nitrogen in vegetative plant parts (Foulkes et al. 2009), nitrogen should not be applied in excessive or insufficient amounts (Page and Feller 2013). In the study, only a small amount of N was applied to the plants through ebb-and-flood irrigation, thus preventing deficiency and oversupply of nitrogen-derived nutrients. In general, the process water was classified as “slightly saline”, with values ranging between 0.7 and 2.0 dS/m (Table 2). Higher values have been associated with reductions in plant height, number of tillers, lower chlorophyll content, and root degeneration (Thapa et al. 2017; Shah et al. 2017; Kulshreshtha et al. 2022). However, these effects were not observed, indicating adequate nutrient conditions in the IAU despite a reduced EC value than control but with qualitatively more biologically derived nitrogen ions.

The light conditions in the greenhouse, determined by sunlight, were comparable between the groups in terms of photosynthetic photon flux density (PPFD, overall mean ≈ 575 µmol/m^2^s; Table 2). *T. aestivum* responds differently to light energy during the cultivation phase, whereby the need for light increases. Dong et al. (2014) reported better shoot length during the first 8–10 days under low light conditions of 200–250 µmol/m^2^s (LED: white 50%, red 50%) with a subsequent growth restriction from day 10 to day 20 compared to initial high light conditions of 425–475 µmol/m^2^s. This result was confirmed by Page and Feller (2016), where plants showed higher dry weights and larger leaves at 380 µmol/m^2^s (vs. 100 µmol/m^2^s) when grown for ≈ 17 DAS. In our study, the natural light input was dependent on the position of the sun, cloud formation and the greenhouse construction and was on average considerably higher than under artificial low light conditions. However, the light energy showed a wide variance e.g. in the case of the IAU group (Table 2), with the minimum light energy being only half the value reported by Dong et al. (2014) under low light conditions. Despite strongly fluctuating light energy, shoot growth in the C/V + IAU substrate was about 34% higher (Table 5) than the 15 cm reported by Dong et al. (2014) for the same duration. The reason for this is not fully understood, but it may be due to a combination of optimal temperature (Thies et al. 2024), light supply, nutrient availability and microbial growth promoters that have not yet been identified. In general, greenhouse cultivation can show advantages, as reported by Dong et al. (2015) on elongation of the stem axes when *T. aestivum* was planted on sandy loam alluvial soil and cultivated under sunlight. In contrast, cultivation under controlled conditions resulted in a reduction of plant length, but at the same time, better yields and a higher thousand-grain weight were observed when plants were grown under artificial light of 500 µmol/m^2^s over 24 h (LED: white 20%, red 80%) and CO_2_ fertilisation (Dong et al. 2015). The PPFD in the study was relatively high but still below the value of 600 µmol/m^2^s that was indicated as the threshold for photoinhibition in the wheat three-leaf stage (Li et al. 2017). Thus, PPFD levels were within the ranges reported in the literature and acceptable for the growth of *T. aestivum* with high light variability due to the “half-open” experimental conditions in the greenhouse.

#### Plant growth performance

Process water of *C. gariepinus* production from the IAU showed a promoting effect on plant height and shoot length in both substrates (Tables 4 and 5) and even showed more advanced growth parameters than the control with Einheitserde substrate (Fig. 6). In general, the growth height of wheatgrass was greater than reported in other experiments under non aquaponic conditions during around the same growing period. Lae and Oo (2014) found a 2.4-fold lower plant height of *T. aestivum* in Myanmar, which was 15.0 cm at day 11, cultivated in soil and water. Furthermore, Dong et al. (2014) reported a 1.4-fold shorter shoot height of approx. 15 cm after 10 days of cultivation in vermiculite pots supplied with a modified Hoagland solution. In contrast, Xu et al. (2022) found a greater height of 46.78 cm in a substrate mixture of 70% coconut fibre and 30% perlite when cultivated under the same aquaponic conditions for a 6-day longer experiment duration. Thus, the aquaponic solution derived from IAU wastewater, with its specific organic nutrient composition, resulted in a substantially greater plant growth performance, even compared to the group with fertiliser and Einheitserde substrate (Fig. 6). Determining factors, except for the high nitrate content in the IAU (Table 2), remain unknown; however, an influence of microorganisms is suspected and should be investigated in future studies.

No differences were found in root length and root dry weight between the substrates (Figs. 3, 4 and 5) as an indicator of sufficient nutrient supply in aquaponic groups and the C/V substrate. In general, the root lengths of *T. aestivum* were comparable to wheat grown under non–water stress conditions, at around 14 cm (Tables 4 and 5), while under water stress conditions (artificially induced by chemical PEG 6000) root growth decreased by about half (Ayalew et al. 2015). On nutrient–poor soils, plants can have a fivefold higher root-to-shoot length ratio (due to root elongation) compared to hydroponics (Chapin 1980), not found in the present study. Besides, C/V substrate combined with water from IAU showed an increase ≈ 16% in root wet weight biomass compared to Einheitserde with the same root length (Fig. 5), which indicates an expansion of the root mass to enhance nutrient uptake under adequate nutrient supply. Therefore, process water from the IAU can be recommended for the cultivation of *T. aestivum*, with the C/V substrate supporting plant growth by providing additional nutrients.

Relative chlorophyll content (SPAD) of wheat was increased by IAU process water in combination with the C/V substrate (IAU > C; Table 5). Higher SPAD levels were found in winter wheat 18 days after germination with approx. 45% at low light exposure (100 µmol/m^2^s) and with around 52% at high light illumination (380 µmol/m^2^s) cultivated with an artificial nutrient solution (Page et al. 2012). Furthermore, the SPAD value of *T. aestivum* showed considerable variations under different water stress conditions. Greenhouse cultivation in Iran demonstrated SPAD values of 30.4–46.2% with good irrigation, 31.3–38.4% with moderate irrigation—comparable to the present study, and 1.2–4.1% under drought conditions (Fotovat et al. 2007). In contrast, field cultivation in Jordan showed higher values at different growth stages from flag leaf emergence out to 52% (GS69, Al-Ghzawi et al. 2018). However, Xu et al. (2022) reported lower SPAD values of 28.17% in the IAU compared to control (27.60%) and EAU (27.29%) under the same experimental conditions. In the present study, *T. aestivum* was only grown for a short time until the three-leaf stage, which according to the AHDB (2022), corresponds to a stage of “GS13” with obviously reduced chlorophyll content compared to mature plants. Furthermore, the higher SPAD content in the C/V + IAU substrate could be explained by the higher iron value in the IAU effluents and to a greater extent in the dry matter of the C/V substrate (Tables 2, 3 and 5). Thus, the use of the C/V substrate in combination with process water from IAU can be recommended for the cultivation of young *T. aestivum*.

Composition of leaf crude nutrients in the C/V substrate was not affected by the irrigation solutions (Table 6) and was comparable to the subsequent wheat cultivation (SC[IAU]) except for ash, which was greater due to a higher EC value. Moisture content was only slightly higher than 83.40% as reported by Kalra et al. (2018; Table 6) for 7-day-old wheat, while the protein content was in the range from 2.69% to 3.34% as described by Xu et al. (2022) for wheat cultivated for additional 6 days. Furthermore, lipid content fell within the range from 0.50% to 0.70%, while ash (1.33–1.54%) and crude fibre content (1.50–2.10%; Xu et al. 2022) were slightly higher in the present study and were within the levels reported in the literature.

Irrigation groups showed no effect on the leaf content of magnesium, sulphur, sodium, copper, iron, zinc, and molybdenum in *T. aestivum* cultivated in the C/V substrate (Table 6), which indicates an adequate nutritional value of wheatgrass when grown under aquaponic conditions. The subsequent cultivation (SC, *Analysis II*) of wheatgrass in the IAU showed slightly higher values for calcium, sodium and zinc, which can be explained by the higher EC value in the SC but basically confirms the observed values of the first examination.

Nitrogen content in the leaves was positively influenced by IAU process water in combination with the C/V substrate (IAU > C, Table 6). The higher *N*-value in the IAU can be explained by the 3.7-fold higher stocking density of *C. gariepinus* (feed N input), which was 1.7-fold greater in the process water for NO_3_^−^ than in control and 2.6-fold higher than in the EAU (Table 2). Although the C/V substrate had a 2.8-fold lower organic N content compared to Einheitserde (Table 3), there was a quantitative compensation of nitrogen through the liquid nutrient additions from the IAU (Table 2), which increased the total nitrogen content in *T. aestivum* (Table 6). Nitrogen deficiency in wheat can have negative effects on plant height, tillering, flag leaf area, and total nitrogen content (Curci et al. 2017), which were not observed in the study, specifically in the IAU plants.

Total phosphorus content was comparable between aquaponic groups (Table 6), even though the PO₄^3^⁻ content in the IAU process water was about 1.8-fold higher than in the EAU (IAU > EAU; Table 2). Phosphate deficiency in wheat showed increased root length, decreased total P content (Römer et al. 1988), and reduced shoot and root biomass (Liao et al. 2008). Since leaf P content in control was higher by about half (Table 6), phosphorus may not be a relevant factor for the reduction in shoot dry weight in the C/V substrate (Table 5). In general, the C/V substrate provided a basal supply of phosphate (18-fold higher than in Einheitserde; Table 3), which, together with the second highest process water P content in the IAU (Table 2), promoted the growth of *T. aestivum*. As there were no signs of P deficiency in IAU plants, an adequate P supply can be assumed.

Potassium was not different between aquaponics groups (Table 6) and approx. fivefold higher compared to 15-day-old wheatgrass cultured in organic growth medium “black cotton soil” with a K content of 13,821 mg/kg (Kulkarni et al. 2006). In C/V substrate, K was about 6.4-fold higher than in Einheitserde (Table 3), indicating a sufficient K reservoir for the requirements of *T. aestivum*. Despite the approximately 17.1-fold lower K content in the IAU process water compared to control (Table 2), the IAU group showed the best plant performance in the C/V substrate (Table 5), indicating that no K deficit can be assumed. Potassium deficiency can result in reduced growth due to decreased photosynthetic carbon assimilation (Rawat et al. 2022), reduced biomass and impaired root development (Thornburg et al. 2020). Shoot dry weight was reduced in control and EAU plants, but not in the IAU group (Table 5), suggesting that IAU process water could be successfully combined with C/V substrate.

Calcium was increased in the plant leaves of the IAU compared to control (Table 6) influenced by repeated water exchange rates of the IAU due to high fish stocking density. Increased water changes in IAU resulted in the accumulation of Ca due to naturally high limestone resources (calcium carbonate) in the process water (water from the local river “Warnow”) along with the feed-derived Ca input, whereby the relatively high Ca content in the C/V substrate served as a base reservoir (Table 3). However, symptoms of calcium deficiency such as lower shoot length and lower fresh weight (Arshad et al. 2012) were observed in the control and EAU (Table 5), where the Ca process water content was lower (Table 2). In contrast, the Ca content in the leaves of the IAU was comparable to that reported by Kulkarni et al. (2006) at 2257 mg/kg, with no deficiency symptoms. Consequently, in the case of Ca, the combination of C/V + IAU substrate can be recommended for wheatgrass cultivation, considering the importance of the local chemical water composition.

Boron levels in plants were comparable in aquaponic groups and higher in the fertiliser solution (C > EAU = IAU; Table 6). Boron (B(OH)_3_) functions as a micronutrient in vascular plants e.g. in nucleic acid stimulation and enzyme activation (Marschner 1995; Sonnewald 2021; Kulshreshtha et al. 2022). Wheat is relatively insensitive to boron deficiency in the young stage, but symptoms such as longitudinal splitting of newer leaves, development of a sawtooth effect at the edges of young leaves and reduced root growth may occur (Kumar et al. 2015; Rerkasem and Jamjod 2004), and were not observed in the study. The threshold for boron deficiency in the young stage was reported as > 1.0 mg/kg in the dry matter, which was exceeded by a factor of 0.7 in Einheitserde and by a 3.5-fold in the C/V substrate (Rerkasem and Jamjod 2004; Table 3). For adequate growth of wheat, boron can be applied via the C/V substrate (Table 3) to achieve sufficient quantities in relation to the fertiliser B content of the C/V + control substrate group.

Manganese levels were comparable in the leaves of aquaponic groups and higher in control (C > EAU = IAU, Table 6). Mn content was determined by fish feed and probably by the natural Mn content of the seeds, which was described by Plaza et al. (2003) as relatively high at 34 mg/kg, along with a high Mn content in the C/V substrate (Table 3). One third of the theoretical total Mn content (seeds + feed + substrate = 151.3 mg/kg Mn) was found in the leaves of IAU plants (Table 6), which almost reached the proportion of the fertiliser group and highlighted the importance of the substrate-induced Mn supply. However, the 3.6-fold higher feeding rate of the IAU (Table 1) had no influence on the Mn content in the leaves compared to the EAU (Table 6), suggesting only a limited impact of feed-derived Mn. In general, Mn levels in the IAU were 2.7-fold higher than 18.7 mg/kg reported by Kulkarni et al. (2006) for wheatgrass cultivated in black cotton soil. A concentration of 50 mg/kg Mn is considered to be sufficient for optimal growth (Kirkby 2012), a value that was nearly reached in the aquaponic groups (Table 6). The threshold for Mn deficiency was reported to be between 10 and 20 mg/kg (Broadley et al. 2012), which was greatly exceeded in the study. Manganese plays a key role in photosynthesis; however, excessive Mn levels can impair photosynthetic efficiency and reduce biomass production (Millaleo et al. 2010; Sonnewald 2021). Thus, the total manganese content was sufficient for *T. aestivum*, mainly due to the high proportion in the C/V substrate.

Iron content was comparable between all groups (Table 6) and exceeded the value of 68.5 mg/kg reported by Kulkarni et al. (2006) for 10-day-old wheatgrass cultivated in the organic medium “black cotton soil”. Fe deficiency in *T. aestivum* (10 days old) is characterised by decreased root growth, decline in both fresh and dry shoot biomass, leaf chlorosis, and interveinal yellowing (Hua et al. 2022). IAU plants cultivated in the C/V substrate showed the best parameters of chlorophyll (SPAD) and growth (Table 5). These improved performance values were determined by the Fe^2^⁺ content in the IAU process water, as well as the high iron content in the C/V substrate (Tables 2 and 3). For other plants with higher nutrient demands, such as basil (*O. basilicum*), Fe has to be continuously supplied in chelated form under aquaponic conditions (Ferrarezi and Bailey 2019), which is evidently not necessary for *T. aestivum*.

Zinc amount was about five-fold higher (Table 6) than reported by Kulkarni et al. (2006) at 6.8 mg/kg for 10-day-old wheatgrass. Wheat is the most sensitive of the cereal plants to Zn deficiency, and the critical level for young wheat leaves has been reported as 14 mg/kg d.m. (Brennan 2001; Rehman et al. 2018), which was exceeded by a factor of about 2.3 in this study (Table 6). Theoretically, about one third of the Zn from the feed and the C/V substrate (Table 3) was found in the leaves of EAU and IAU (Table 6). Deficiency of Zn can decrease shoot length, leaf size and chlorophyll content (Cakmak et al. 1998; Rehman et al. 2018). In the study, a higher SPAD value and better growth was observed in the IAU group compared to control in the C/V substrate (Table 5). Thus, the high Zn content of the C/V substrate (Table 3), together with the zinc content of the feed, provided a sufficient amount for the growth of *T. aestivum* under aquaponic conditions.

To summarise, the proportion of the individual minerals depends in large part on the availability of nutrients in the growth medium (C/V substrate), the quantity in the feed, the nutrient quality of the irrigation water and the age of plants. Since no crude nutrient and mineral deficiency occurred in this study, it can be assumed that aquaponic production of *T. aestivum* with *C. gariepinus* is possible without loss of plant quality. However, the alternative C/V substrate can still be optimised. Vermiculite is known for its good buffering properties for the heavy cations Cu^2+^, Co^2+^ (Da Fonseca et al. 2005), potassium and magnesium (Swain et al. 2021), which were also present in higher proportions in the C/V substrate (Table 3). For a complete substitution of Einheitserde, a further fraction, consisting of humus material with higher proportions of organic components (2.2-fold), in particular nitrogen (2.8-fold) and sulphur (3.8-fold; Table 3), would have to be added to the C/V substrate. Furthermore, for optimised nutrient soil properties and plants with higher quantitative nutrient requirements (Somerville et al. 2014), the following substrate mixture should be tested: humus fine 35% (Hf) as a general nutrient store for organic substances (e.g. N, P, amino acids), humus coarse 15% (Hc) as a coir fibre substitute (or bark compost after Eymann et al. 2015), vermiculite 25% (V) as a store for cations (e.g. K, Mg, Cu) and montmorillonite-containing clay 25% (M) as a store for NH_4_^+^ (Nieder et al. 2011; Knaus et al. 2024) i.e. basically a four-fraction substrate (Hf, Hc, V, M). The organic matter fraction (e.g. humus) should originate from a circular economy to enhance the ecological value of the substrate mixture.

#### Vitamin content

In general, vitamin content showed no differences between irrigation with fertiliser and aquaculture process water and was therefore comparable, except for vitamin B_6_, which was greater in the leaves of the IAU group plants (IAU > C = EAU; Table 6).

The higher content of vitamin B_6_ in IAU is unclear but does not appear to be coincidental. Higher levels of Vitamin B_6_ were found in a former experiment with the same experimental conditions and 100% coconut substrate in the FGH with a level of 1.42 mg/100 g (calculated according to d.m.) in the IAU, when the growth period was extended by 6 days (Xu et al. 2022). Qamar et al. (2018) reported slightly higher amounts of B_6_ with 0.96 mg/100 g where wheatgrass appeared to be some days older (approx. four days). The occurrence of vitamin B_6_ in wheatgrass leaves seems to increase with plant age. As a cofactor of protein biosynthesis, B_6_ could accumulate with increased plant height, which would explain the relatively low concentrations in the present study in younger plants.

The vitamin B_6_ content in the leaves of *T. aestivum* was calculated as the sum of the derivatives (pyridoxal, pyridoxamine, pyridoxine), whereby the significant result is due more to deviations of pyridoxine in the IAU (Table 6). As all B_6_ derivatives have at least one nitrogen atom in the molecule, there appears to be a correlation with the levels of N-based substances in the process water. Robinson et al. (2016) reported a possible dependence of B_6_ biosynthesis on nitrogen-based substances such as NH_3_. In wild-type *Arabidopsis* spp., a similar effect was described with higher B_6_ levels when plants were fertilised with ammonium chloride (NH_4_Cl) and ammonium nitrate (NH_4_NO_3_), as opposed to a nitrogen-free nutrient solution (Colinas et al. 2016). The higher content of B_6_ seems to correlate positively with N-derived chemical water compounds (NH_4_^+^, NO_2_^−^ and NO_3_^−^; Table 2), which was higher as a sum in IAU compared to EAU by 61.4% and in the control by 39.6%.

Vitamin B_6_ is essential for humans and animals and has to be supplied from plant sources, which are able to synthesise B_6_ de novo (Vanderschuren et al. 2013). Water soluble Vitamin B_6_ consists of six different forms: pyridoxal, pyridoxamine, and pyridoxine, including their phosphoric acid derivates (Habermehl et al. 2008; Vanderschuren et al. 2013; EFSA Panel 2016; Lal 2018). The content of pyridoxine in wheat varied between the years and the variety, and sometimes by laboratories or methods (Keagy et al. 1980; Davis et al. 1981). The role of B_6_ in plants is mainly in amino acid synthesis and catabolism, but also in sugar and fatty acid metabolism, especially as a cofactor of metabolic enzymes (Genevois 1964; Marschner 1995; Hellmann and Mooney 2010; Vanderschuren et al. 2013). In common wallcress (*Arabidopsis* spp.), the lack of B_6_ showed impaired seed development, reduced growth, and oxidative stress (Vanderschuren et al. 2013). In humans, the biologically active form is pyridoxal 5′–phosphate (PLP) and functions in the metabolism of amino acids, neurotransmitters, lipid and carbohydrate assembly and erythropoiesis (Lheureux et al. 2005; Wilson et al. 2019). Symptoms of deficiency are wide ranging, such cardiovascular diseases, epilepsy, anaemia, depression, Alzheimer’s disease, while in contrast B_6_, showed positive effects against cancer and acts as an antioxidant (Hellmann and Mooney 2010; Vanderschuren et al. 2013; Wilson et al. 2019). The vitamin B_6_ requirement for adults was specified as 1.4 mg/day for women and 1.6 mg/day for men (Jungert et al. 2020; German Nutrition Society 2022a), with the values differing slightly (women: 1.3 mg/day; men: 1.5 mg/day; EFSA Panel 2016). The content of B_6_ found in the IAU (0.78 mg/100 g) was about 50–60% of the daily nutritional requirement, and thus, extraction would be recommended as a dried food supplement in powder form or tablets.

Levels of folic acid (B_9_), vitamin B_12_ and vitamin E were not significantly different and showed that production of *T. aestivum* in aquaponics is possible without reduced quality in vitamin content (Table 6). For folic acid, Qamar et al. (2018) reported significantly 94% lower content (≈ 16-fold) with 84.0 μg/100 g compared to the mean amount found in the IAU. For adults, a daily intake of 300 µg as dietary folate has been recommended, the observed amount was more than fourfold of the daily nutritional requirement (Krawinkel et al. 2014; German Nutrition Society 2022b). Generally, folic acid (B_9_; former B_4_, also named B_11_; van Dusseldorp 1997; Liew 2016) influences cell growth and division as well as the maintenance of cardiovascular health and has preventive effects against cancer, depression, and dementia (Eichholzer et al. 2006; Lensch et al. 2011). One daily juiced ration from ≈ 22 g wheatgrass would cover the requirement for human nutrition of folic acid.

The level of vitamin B_12_ was substantially lower (Table 6) than reported by Qamar et al. (2018) with 66.87 μg/100 g in *T. aestivum*. Vitamin B_12_ (cobalamin) is essential for humans and can only be synthesised by prokaryotes. The B_12_ content in the EAU and IAU probably originated from microorganisms that were present in the process water of the aquaponics. For an adequate intake of 4.0 µg/day B_12_ for adults (Ströhle et al. 2019), the content in the EAU was slightly lower (96.5%). B_12_ is generally effective in preventing various symptoms of malnutrition such as megaloblastic anaemia and neuropathy, with a focus on vegetarian diets (Watanabe 2007; Hunt et al. 2014).

Vitamin E was substantial higher (EAU: 3.14 mg/100 g = 31.4 µg/g, Table 6) than reported in 14-day-old *T. aestivum* with 0.228 µg/g α–tocopherol cultured in pots with soil and peat (Karakas et al. 2021). Tocopherol biosynthesis occurs in the photosynthetic tissue of plants, has antioxidant effects, protects the chloroplast membrane and polyunsaturated fatty acids (PUFA) from peroxidation (Hussain et al. 2013), and can vary by plant age, organs, and hormones (Hasanuzzaman et al. 2014). For adult humans, a daily intake of 12–15 mg/day is indicated and protects against oxidation of membrane lipids, acts against cancer, arthritis, circulatory diseases, and diabetes (Schwarzova et al. 2021). To achieve a daily ration of vitamin E for humans, the amount would have to be increased fivefold.

## Conclusions

Wheatgrass (*Triticum aestivum*) showed the best growth performance when irrigated with process water from the IAU production of *Clarias gariepinus* in both substrates. The growth-enhancing effect of IAU process water was more pronounced in the C/V substrate, with seven significantly improved parameters compared to the fertiliser control group and five compared to the EAU group. In contrast, no growth difference was observed between C/V + EAU plants and the fertiliser group, which simultaneously supports the use of process water from extensive aquaculture production. The growth of wheatgrass in the C/V substrate consequently followed the order: IAU > EAU = control. Overall, the differences in growth parameters between IAU and EAU were small and often slightly exceeded the significance threshold (e.g. shoot length), with partially high variances. Tendentially, the C/V substrate showed an improvement in the growth parameters of *T. aestivum* with the use of aquaponic nutrient solutions. Most growth parameters showed higher values in the C/V + IAU substrate (10 of 14) compared to Einheitserde with fertiliser (E + control) and even compared to the C/V + EAU substrate, which indicates an improved nutrient supply in the process water from the intensive production of *C. gariepinus* combined with the C/V substrate.

Different irrigation groups had no effect on the leaf crude nutrient content of proteins and lipids in *T. aestivum* cultivated in the C/V substrate, which supports cultivation in aquaponics. Even though the levels of P, K, B, and Mn in the leaves of C/V substrate aquaponics groups were reduced in comparison to the fertiliser group, there was no evidence of a growth deficiency under IAU irrigation. For this, most nutrients were provided by the C/V substrate, which functioned as a nutrient reservoir (e.g. for P, K, Mg, B, Fe, Mn), except for nitrogen, which was provided by the process water from the IAU (and EAU) as a wastewater by-product. Since nutrients, minerals, and vitamins were similar between EAU and IAU, the nutrient content in the C/V substrate under EAU conditions was sufficient for practical cultivation of *T. aestivum* as a plant with low nutrient requirements. Process water from IAU resulted in a quantitative improvement in growth but not in nutrient and vitamin quality. Therefore, in the case of intensive *C. gariepinus* production, the number of cultivated plants could be increased. Only a higher vitamin B_6_ content was observed in plants from the C/V + IAU substrate group, whose origin was unclear; however, a correlation with higher N applications could not be excluded. Folic acid (vitamin B_9_) was available in sufficient quantities for human consumption and could possibly be obtained in larger quantities as fresh juice. With an increase in plant production, the formulation of dried vitamin powder or tablets seems possible and could be used as a dietary supplement. The study specifically addressed high nitrogen loads at high stocking densities of *C. gariepinus* production (IAU). Future studies should aim to reduce nitrate levels while increasing ammonium concentrations by varying fish species and stocking densities, which could be beneficial for other plant species, such as herbs.

Generally, standard growing media “Einheitserde” can be replaced by a coconut and vermiculite substrate (50:50) combined with process water from *C. gariepinus* production to reduce environmental impact from methane and CO₂ emissions associated with peat extraction. Under practical conditions, process water from extensive *C. gariepinus* production can be successfully used to provide sufficient nutrient ratios, as effluents from intensive production only improved plant growth parameters. In addition, a positive influence of microorganisms as promoters of plant growth and vitamin synthesis (e.g. vitamin B_12_) was suspected. However, the possibility of harmful bacteria in the aquaponic process water cannot be excluded and the risk of human contamination remains as a subject for future investigations. Beyond that, this study demonstrated a previously less explored approach to mitigating the impact of potentially harmful microorganisms in aquaponics, focusing on the commercial use of a plant-based product. Microorganisms can be eliminated by the procedures of extraction and drying of secondary plant metabolites (e.g. minerals and vitamins) for further harmless use as dietary supplements.

Coconut fibre itself is a component of the agricultural circular economy; however, their cultivation should not be expanded unreservedly due to competition with natural tropical forests, as palm groves are often planted in monoculture. Therefore, the coconut content of the C/V substrate should be reduced in the future. An alternative substrate mixture can be recommended, consisting of four fractions: humus (fine, coarse), vermiculite, and montmorillonite whose exact proportions should be determined. The humus or compost fractions should originate from agricultural circular economy processes to ensure sustainable aquaponic production.

## Data Availability

All data obtained or analysed as part of this study are included in this published article.
